# Anti-Inflammatory Activities of Pentaherbs Formula, Berberine, Gallic Acid and Chlorogenic Acid in Atopic Dermatitis-Like Skin Inflammation

**DOI:** 10.3390/molecules21040519

**Published:** 2016-04-20

**Authors:** Miranda S. M. Tsang, Delong Jiao, Ben C. L. Chan, Kam-Lun Hon, Ping C. Leung, Clara B. S. Lau, Eric C. W. Wong, Ling Cheng, Carmen K. M. Chan, Christopher W. K. Lam, Chun K. Wong

**Affiliations:** 1 Institute of Chinese Medicine, The Chinese University of Hong Kong, Hong Kong, China; tsangsinman0128@gmail.com (M.S.M.T.); benchan99@cuhk.edu.hk (B.C.L.C.); ehon@cuhk.edu.hk (K.-L.H.); pingcleung@cuhk.edu.hk (P.C.L.); claralau@cuhk.edu.hk (C.B.S.L.); cwwong_eric@cuhk.edu.hk (E.C.W.W.); b750741@mailserv.cuhk.edu.hk (L.C.); ckmchan@cuhk.edu.hk (C.K.M.C.); 2 State Key Laboratory of Phytochemistry and Plant Resources in West China, The Chinese University of Hong Kong, Hong Kong, China; 3 Department of Chemical Pathology, The Chinese University of Hong Kong, Hong Kong, China; j.dl@foxmail.com; 4 Department of Paediatrics, The Chinese University of Hong Kong, Prince of Wales Hospital, Shatin, NT, Hong Kong, China; 5 Li Dak Sum Yip Yio Chin R & D Centre for Chinese Medicine, The Chinese University of Hong Kong, Hong Kong, China; 6 State Key Laboratory of Quality Research in Chinese Medicine, Macau Institute for Applied Research in Medicine and Health, Macau University of Science and Technology, Macau, China; wklam@must.edu.mo

**Keywords:** allergic inflammation, atopic dermatitis, berberine, Gallic acid, pentaherbs formula

## Abstract

Atopic dermatitis (AD) is a common allergic skin disease, characterized by dryness, itchiness, thickening and inflammation of the skin. Infiltration of eosinophils into the dermal layer and presence of edema are typical characteristics in the skin biopsy of AD patients. Previous *in vitro* and clinical studies showed that the Pentaherbs formula (PHF) consisting of five traditional Chinese herbal medicines, Flos Lonicerae, Herba Menthae, Cortex Phellodendri, Cortex Moutan and Rhizoma Atractylodis at *w*/*w* ratio of 2:1:2:2:2 exhibited therapeutic potential in treating AD. In this study, an *in vivo* murine model with oxazolone (OXA)-mediated dermatitis was used to elucidate the efficacy of PHF. Active ingredients of PHF water extract were also identified and quantified, and their *in vitro* anti-inflammatory activities on pruritogenic cytokine IL-31- and alarmin IL-33-activated human eosinophils and dermal fibroblasts were evaluated. Ear swelling, epidermis thickening and eosinophils infiltration in epidermal and dermal layers, and the release of serum IL-12 of the murine OXA-mediated dermatitis were significantly reduced upon oral or topical treatment with PHF (all *p* < 0.05). Gallic acid, chlorogenic acid and berberine contents (*w*/*w*) in PHF were found to be 0.479%, 1.201% and 0.022%, respectively. Gallic acid and chlorogenic acid could suppress the release of pro-inflammatory cytokine IL-6 and chemokine CCL7 and CXCL8, respectively, in IL-31- and IL-33-treated eosinophils-dermal fibroblasts co-culture; while berberine could suppress the release of IL-6, CXCL8, CCL2 and CCL7 in the eosinophil culture and eosinophils-dermal fibroblasts co-culture (all *p* < 0.05). These findings suggest that PHF can ameliorate allergic inflammation and attenuate the activation of eosinophils.

## 1. Introduction

The prevalence of allergic diseases such as allergic rhinitis, allergic asthma and atopic dermatitis (AD) has been increasing significantly in both developed and developing countries [1,2,3]. AD is one of the most frequent chronic inflammatory skin diseases, affecting up to 25% of children and 1%–3% of adults worldwide [1]. Also named eczema, AD is the most common type of chronic allergic skin disease. The worldwide prevalence of AD is increasing with about 70% of cases occurring before the age of five [4]. AD is a long-lasting skin disorder; patients may suffer from episodic exacerbations and remissions during their lifetime [5]. Typical symptoms of AD include extremely itchy, inflamed and dry skin, the inflamed area can be red, swollen, cracked, scaled, webby and crusted [5,6].

There is no definitive cure for AD and the current effective treatment involves topical application of immunosuppressive steroids with undesirable side effects. Therefore, there has been a rising interest in the development of safer and non-steroid immunomodulatory formulas for the treatment of AD. Traditional Chinese Medicine (TCM) has become a more accepted and increasingly used modality for immunomodulation. Our previous clinical trial has shown that the quality of life of children with manifested moderate-to-severe AD was significantly improved after a 12-week treatment with TCM Pentaherbs formula (PHF), concurrently with reduced use of topical corticosteroids [7]. Such finding suggested that PHF could potentially be an alternative adjunct therapy for AD. PHF contains five herbs: Flos Lonicerae (Jinyinhua), Herba Menthae (Bohe), Cortex Moutan (Danpi/DP), Rhizoma Atractylodis (Cangzhu), and Cortex Phellodendri (Huangbai) at a *w*/*w* ratio of 2:1:2:2:2 [7]. It has been further formulated to investigate its cellular mechanisms for anti-inflammatory activities. PHF with an increased ratio of Danpi exhibited a greater inhibitory effect on the release of the inflammatory cytokines interleukin (IL)-6 and IL-1 from HMC-1 human mast cells [8]. Gallic acid (GA, 3,4,5-trihydroxybenzoic acid), a potent anti-oxidant found in witch hazel and tea leaves, is one of the main ingredients in Danpi [9]. GA has been shown to possess anti-tumorigenic effect and anti-inflammatory activity in both nude mice and human cell lines [10,11]. We have shown that PHF, Danpi and GA can exhibit anti-allergic inflammatory activity on human basophils (KU812 cells) and human monocyte-derived dendritic cells, which are crucial effector cells in allergic inflammation [12,13]. Another active ingredient of Cortex Phellodendri, berberine, has recently been shown to reduce allergic inflammation in a house dust mite-induced allergic rhinitis mouse model [14].

IL-33 is a novel member of the IL-1 cytokine family that is released passively during cell necrosis and tissue damage [15,16,17]. It has been characterized as a potent pro-inflammatory Th2 cytokine that acts on immune cells such as mast cells, eosinophils, basophils, and type 2 innate lymphoid cells in allergic inflammation [15,16,17]. In addition, the inflamed skin lesions of AD patients have both elevated protein and mRNA expression of IL-33 compared to normal subjects [15,17]. IL-31 is the pruritogenic cytokine in AD that has been shown to activate eosinophils interacting with skin cells [15,18].

The histology of AD is characterized by dermal and epidermal inflammatory infiltrates including eosinophils [19]. Eosinophil infiltration and activation have been shown in AD skin lesions [20,21]. Both tissue and blood eosinophilia are features in acute and chronic stages of AD and they were found to positively correlate with disease severity [22,23]. Moreover, eosinophilic granular protein such as eosinophil derived neurotoxin deposition has been found in nearly all biopsies of AD lesions [24,25]. Eosinophil activator IL-5, chemokine CCL5 and eotaxin can activate and mediate the infiltration of eosinophils into the inner dermal fibroblast layer causing inflammation of AD [26].

In an attempt to further elucidate the anti-inflammatory mechanisms of PHF on AD, we have investigated the *in vitro* and *in vivo* anti-inflammatory activities of the PHF and its potential active ingredients on mice with oxazolone (OXA)-induced dermatitis and eosinophils co-culturing with human dermal fibroblasts, respectively.

## 2. Results and Discussion

### 2.1. Effects of Pentaherbs Formula on the Ear Redness of Challenged Ear of OXA-Induced Dermatitis Mice

**Figure 1:**
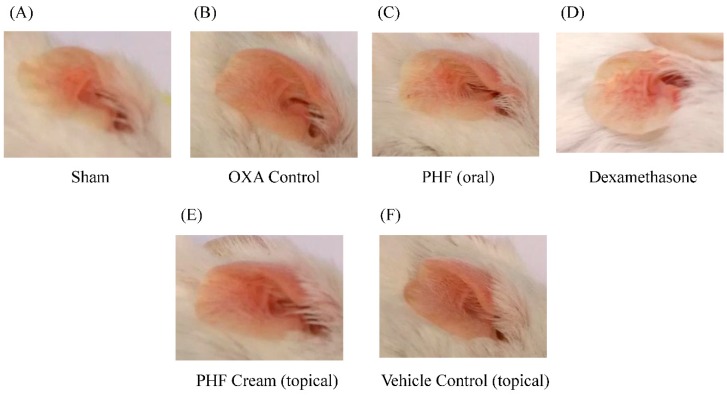
Oral treatment of Pentaherbs formula (PHF) could relieve *in vivo* oxazolone (OXA)-mediated dermatitis of mouse ears. A single challenge OXA-mediated dermatitis murine model was established by administering OXA on mice ears after sensitization. PHF (15 mg) in 0.3 mL PBS was orally administered or topically applied on mice ears from Day 1 to Day 6. Dexamethasone (2.5 mg/mL) was topically applied on mice ears as positive control. (**A**–**F**) Representative photographs of ear redness were taken on Day 9 before mice were sacrificed (*n* = 6).

Our previous study has shown that PHF can improve the quality of life of AD patients and reduce their corticosteroids consumption [7]. However, no *in vivo* evidence has suggested that PHF can ameliorate inflammatory symptoms. Therefore, an *in vivo* murine model using oxazolone (OXA), a hapten that can induce allergic contact dermatitis-like or atopic dermatitis-like skin inflammation, was used to elucidate the anti-inflammatory action of PHF. Chronic allergic contact dermatitis displays clinical, histological and immunologic features that are comparable to atopic dermatitis, while OXA-induced dermatitis could produce AD-like symptoms including epidermal hyperplasia and inflammatory cells infiltration [27]. Figure 1B shows that mouse ears became red and swollen after single OXA challenge on Day 8. After treating the OXA-mediated dermatitis mice with PHF orally for six consecutive days, the redness of the OXA-challenged ear (right ear) was relieved (Figure 1C) as compared to the non-treated OXA control group (Figure 1B). However, the effect of topical treatment of PHF on the mouse ears was not prominent (Figure 1E).

### 2.2. Effects of Pentaherbs Formula on the Ear Swelling of Challenged Ear of OXA-Induced Dermatitis Mice

**Figure 2:**
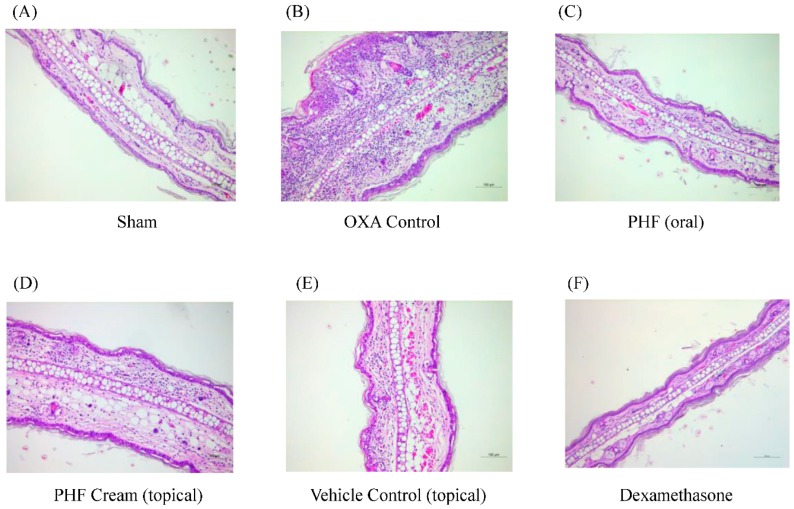
Oral intake of PHF could reduce the ear tissue swelling of mice with OXA-mediated dermatitis. A single challenge OXA-mediated dermatitis murine model was established by administering OXA on mouse ears after sensitization. PHF (15 mg) in 0.3 mL PBS was orally administered or topically applied on mouse ears from Day 1 to Day 6. Dexamethasone (2.5 mg/mL) was applied topically on mouse ears as positive control. (**A**–**F**) Representative hematoxylin and eosin stain (H & E) staining, which denoted the thickness of challenged ear (right ear) tissues, was performed on Day 16 (100×) (*n* = 4).

Ear swelling, or hyperplasia, is a feature of atopic dermatitis. Results of the representative H & E staining (100×) of the challenged ear tissue demonstrated that oral treatment of PHF, but not topical treatment, could reduce ear tissue swelling of the OXA-mediated dermatitis model (Figure 2). Although oral treatment with PHF could alleviate ear swelling, the effects were not as effective as the dexamethasone positive control group.

### 2.3. Effects of Pentaherbs Formula on the Epidermis Thickening of the Challenged Ear of OXA-Induced Dermatitis Mice

**Figure 3:**
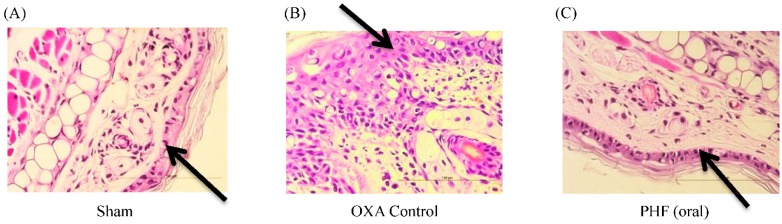

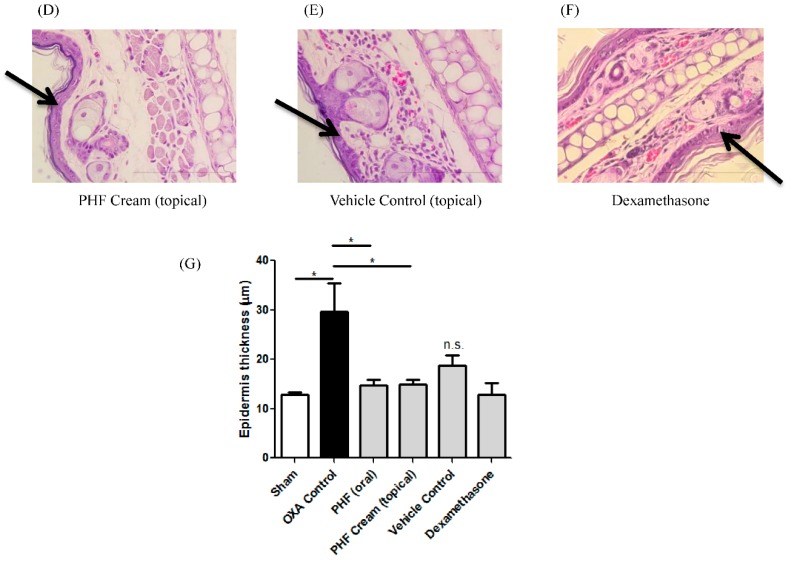
Oral intake and topical application of PHF could significantly reduce the thickness of ear epidermal layer of mice with OXA-mediated dermatitis. A single challenge OXA-mediated dermatitis murine model was established by administering OXA on mouse ears after sensitization. PHF (15 mg) in 0.3 mL PBS was orally administered or topically applied on mouse ears from Day 1 to Day 6. Dexamethasone (2.5 mg/mL) was topically applied on mouse ears as positive control; (**A**–**F**) Representative H & E staining of challenged ear (right ear) was performed on Day 16 (400×). Black arrows depict the epidermal layer of the ear tissue; (**G**) The ear epidermal thickness was measured and shown in bar chart with mean ± SEM (*n* = 6). * *p* < 0.05 when compared with the OXA control group; n.s.: no significant difference when compared with the OXA control group.

Epidermis thickening can be one of the factors that contribute to the ear swelling as mentioned above. In the OXA-mediated dermatitis model, the thickening of epidermis was observed (Figure 3B). After treating OXA-mice with PHF orally or topically, the epidermis thickening was significantly reduced (both *p* < 0.05, Figure 3G). However, topical treatment with drug cream vehicle alone did not significantly reduce the epidermis thickening (Figure 3G, *p* > 0.05), implying that PHF contributes to the anti-inflammatory action. These findings were also consistent with the histology of the challenged ear tissue as observed in H & E staining (400×) (Figure 3A–F).

### 2.4. Effects of Pentaherbs Formula on the Eosinophils Infiltration of the Challenged Ear of OXA-Induced Dermatitis Mice

**Figure 4:**
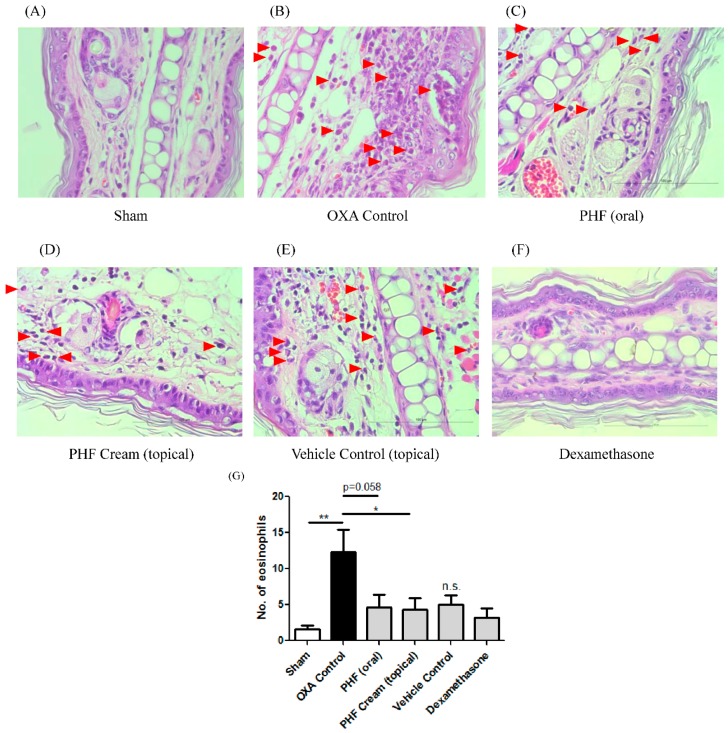
Oral intake and topical application of PHF could reduce the infiltration of eosinophils into the epidermal and dermal layers of mice with OXA-mediated dermatitis. A single challenge OXA-mediated dermatitis murine model was established by administering OXA on mice ears after sensitization. PHF (15 mg) in 0.3 mL PBS was orally administered or topically applied on mouse ears from Day 1 to Day 6. Dexamethasone (2.5 mg/mL) was topically applied on mouse ears as positive control. (**A**–**F**) Representative H & E staining of the epidermal and dermal layers of challenged ear (right ear) was performed on Day 16 (400×). Red arrows point to the infiltrated eosinophils at epidermal and dermal layers; (**G**) The number of eosinophils infiltrated into the epidermal and dermal layers was counted and shown in bar chart with mean ± SEM (*n* = 6). * *p* < 0.05, ** *p* < 0.01 when compared with the OXA control group; n.s.: no significant difference when compared with the OXA control group.

Eosinophilia in cutaneous tissues is a typical feature in both chronic and acute AD patients. In the current OXA-mediated dermatitis model, eosinophils infiltrating into the dermal layer is observed, as denoted by red arrows (Figure 4B). As shown in Figure 4G, oral intake of PHF could marginally reduce eosinophils infiltration into the epidermal and dermal layers (*p* = 0.058). With the topical treatment of PHF on the mouse ears, the number of eosinophils infiltrating into the epidermal and dermal layers of the mouse ears was significantly reduced (*p* < 0.05). Topical treatment with vehicle alone did not significantly reduce the eosinophils infiltration (*p* > 0.05). As eosinophils are responsible to skin inflammation and thickening in allergic skin inflammation [28], the reduction of eosinophils infiltration into the epidermal and dermal layers of PHF-treated mice (Figure 4G) may contribute to the reduction in epidermis thickening, as reported in the previous section (Figure 3G).

The reasons accounting for the non-significant suppressive effect of topical administered PHF on ear edema (Figure 2) may probably be related to the increase in vessel permeability to allow water infiltration to the skin layer by PHF. It is probably due to the physical/biological irritation by the mixed components of PHF. Therefore, the overall ear skin tissue swelling of topical administration of PHF is somehow similar to that of control. Oral intake of PHF will exert anti-inflammatory activity after intestinal absorption and exert the immunomodulation at the local skin area, causing significant suppression of the ear edema, without causing any physical/biological irritation (Figure 2). Apart from the overall ear skin tissue swelling, we found that both oral intake and topical application of PHF could significantly reduce the thickness of ear epidermal layer and the infiltration of eosinophils into the epidermal and dermal layers of mice with OXA-mediated dermatitis (Figure 3 and Figure 4).

### 2.5. Effects of Pentaherbs Formula on the Serum Concentrations of Cytokines and Chemokines of OXA-Induced Dermatitis Mice

**Figure 5:**
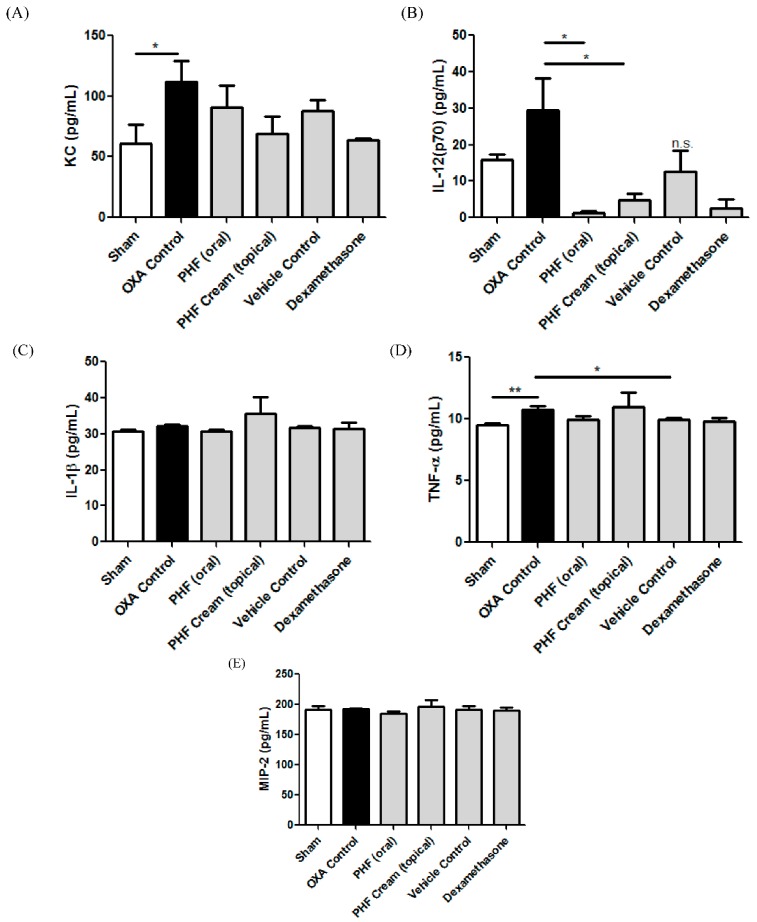
Effect of PHF on the serum concentrations of AD-related inflammatory cytokines and chemokines. A single challenge OXA-mediated dermatitis murine model was established by administering OXA on mice ears after sensitization. PHF (15 mg) in 0.3 mL PBS was orally administered or topically applied on mice ears from Day 1 to Day 6. Dexamethasone (2.5 mg/mL) was topically applied on mice ears as positive control. Serum was obtained on Day 9 after treatments for the determination of concentration of (**A**) KC; (**B**) IL-12 (p70); (**C**) IL-1β; (**D**) TNF-α and (**E**) MIP-2. Results are shown in bar chart with mean ± SEM (*n* = 6). * *p* < 0.05, ** *p* < 0.01 when compared with the OXA control group; n.s.: no significant difference when compared with OXA control group.

Inflammatory cytokine IL-12 (p70) released in blood serum were found to be significantly reduced when PHF was orally administered and topically applied to the ears of the mice (Figure 5B, both *p* < 0.05). However, the reduction of other cytokines and chemokines upon the treatment of vehicle only was not significant (all *p* > 0.05). IL-12, a bioactive heterodimer produced by various immune cell types including dendritic cells, macrophages and neutrophils, is upregulated in AD skin lesions [29,30]. The increase in the release of IL-12 activates cytotoxic lymphocytes, and leads to tissue damage in skin inflammation [30]. The decrease in serum IL-12 concentration in PHF-treated mice indicated PHF might inhibit the activation of dendritic cells, macrophages and neutrophils for the improvement in skin inflammation. However, the detailed cellular mechanisms of PHF-mediated suppressive activity need further investigation.

### 2.6. In Vivo Anti-Inflammatorty Activities of Pentaherbs Formula on Multiple-OXA-Mediated Dermatitis

**Figure 6:**
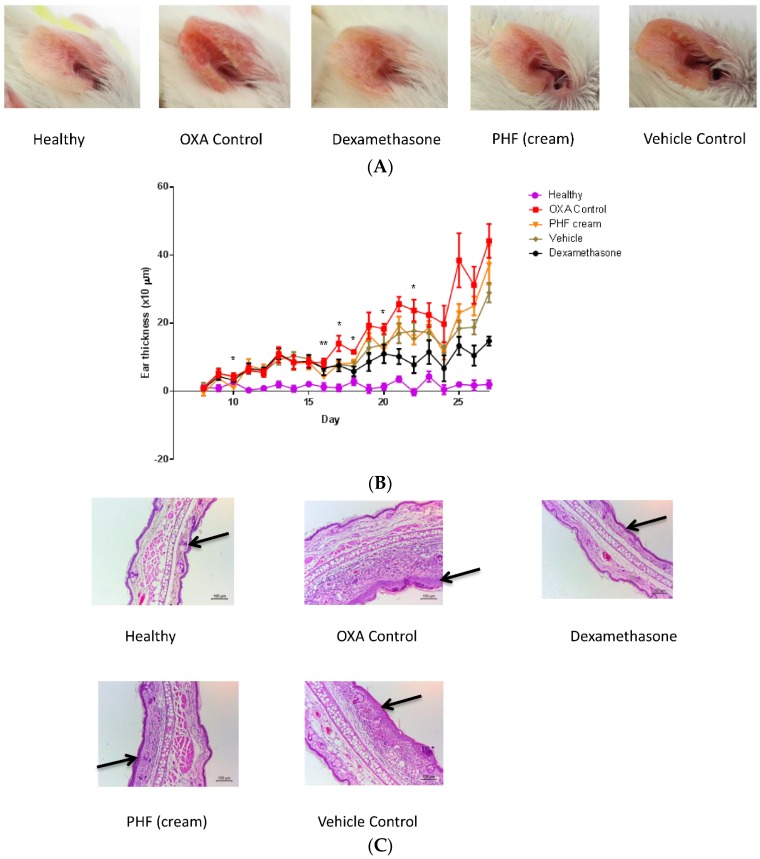

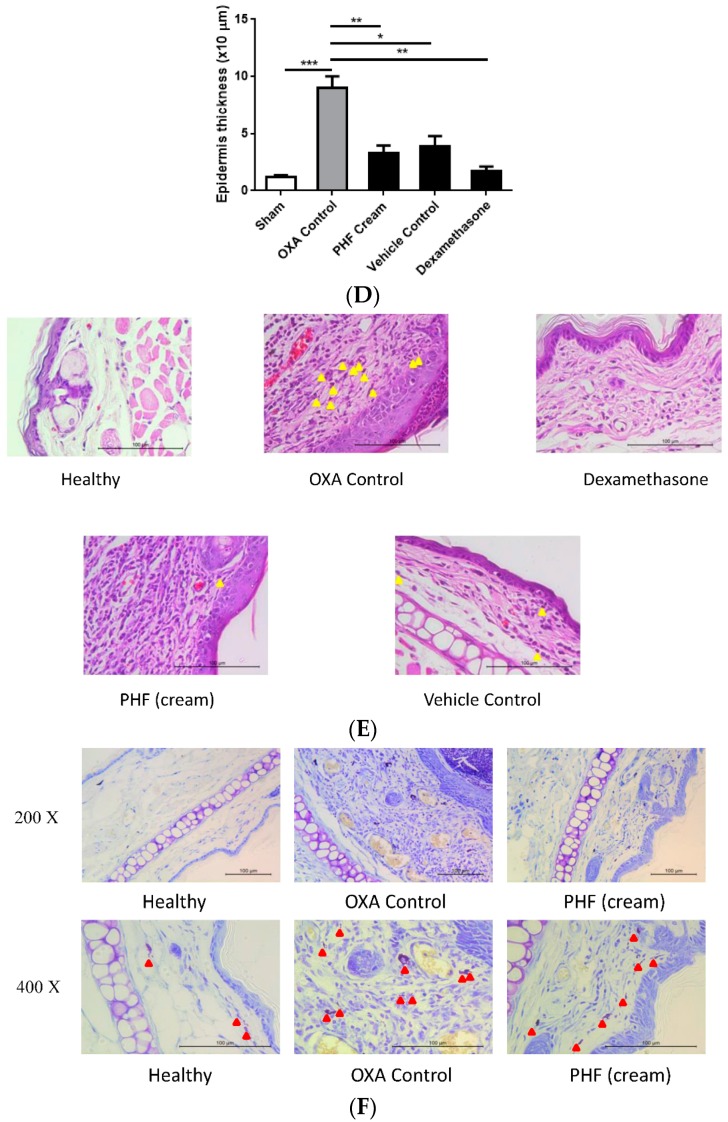
Effects of topical application of PHF on ears of mice with OXA-mediated dermatitis with 10 times OXA challenge. An OXA-mediated AD-like murine model was established by administering OXA on mouse ears ten times after sensitization. PHF (50 mg/mL cream) or cream vehicle (mixture of wool fat and Vaseline) was topically applied on mouse ears from Day 1 to Day 25 every other day. Dexamethasone (10 µg/ear) was applied topically on mouse ears as positive control from Day 16 to Day 26. (**A**) Representative photographs of ear redness of mice with different treatments were taken on Day 27 before mice were sacrificed (*n* = 6); (**B**) The ear thickness was measured and shown with mean ± SEM (*n* = 6) upon different treatments. * *p* < 0.05, ** *p* < 0.01 when comparing the PHF treatment group with the OXA control group; (**C**) Representative H & E staining of the epidermal and dermal layers of challenged ear (right ear) was performed on Day 32 (100×) upon different treatments; (**D**) The ear epidermis thickness was measured using ImageJ, and shown in bar chart with mean ± SEM (*n* = 6). * *p* < 0.05, ** *p* < 0.01, *** *p* < 0.0001 when compared with the OXA control group; (**E**) Representative H & E staining of the epidermal and dermal layers of challenged ear (right ear) of mice was performed on Day 32 (400×) upon different treatments. Yellow arrows point to the infiltrated eosinophils at the dermal layer; (**F**) Representative toluidine blue staining of the epidermal and dermal layers of challenged ear (right ear) of mice with different treatments was performed on Day 32 at 200× and at 400× magnifications. Red arrows point to the infiltrated mast cells at the dermal layer.

Instead of the above single challenge OXA-induced dermatitis, using another mice model with dermatitis-induced by multiple challenge (10 times) with OXA, we found that that serum levels of total IgE and Th2 cytokine IL-4 concentrations in mice were elevated upon the multiple challenges with OXA (mean of serum concentration of total IgE, control: 6.3 ng/mL; OXA: 48.0 ng/mL; mean of serum concentration of IL-4, control: 0.01 pg/mL; OXA: 0.2 pg/mL), thereby indicating the Th2 predomination in such AD mice. Upon the topical treatment by PHF, the OXA-induced ear redness (Figure 6A), thickness of ear (Figure 6B) and ear epidermal layer (Figure 6C,D) and infiltration of eosinophils (Figure 6E) and mast cells (Figure 6F) have been found to be reduced. Moreover, we found the elevation of serum level of eosinophil activator IL-5 upon the multiple challenges with OXA in mice, while the treatments with PHF and dexamethasone suppressed the OXA-induced IL-5 serum level (mean of serum concentration of IL-5, control: 5.5 pg/mL; OXA: 27.8 pg/mL; PHF: 8.4 pg/mL; dexamethasone: 6.5 pg/mL). Results therefore indicated that the PHF treatment could somehow relieve the eosinophils-mediated allergic inflammation for the anti-inflammatory activity in AD. According to the results of the *in vivo* experiments (Figure 6), cream vehicle may sometimes exert certain anti-inflammatory activities in dermatitis. It is because cream vehicle may act as a kind of emollient to retain water and help the external layers of the skin (epidermis) softer and more pliable. Moreover, cream vehicle may protect the skin cells from further damage and disintegration and also facilitate the absorption of active components to penetrate the skin epidermal layer to exhibit its anti-inflammatory activities.

### 2.7. Quantification of Active Ingredients in Pentaherbs Formula

**Figure 7:**
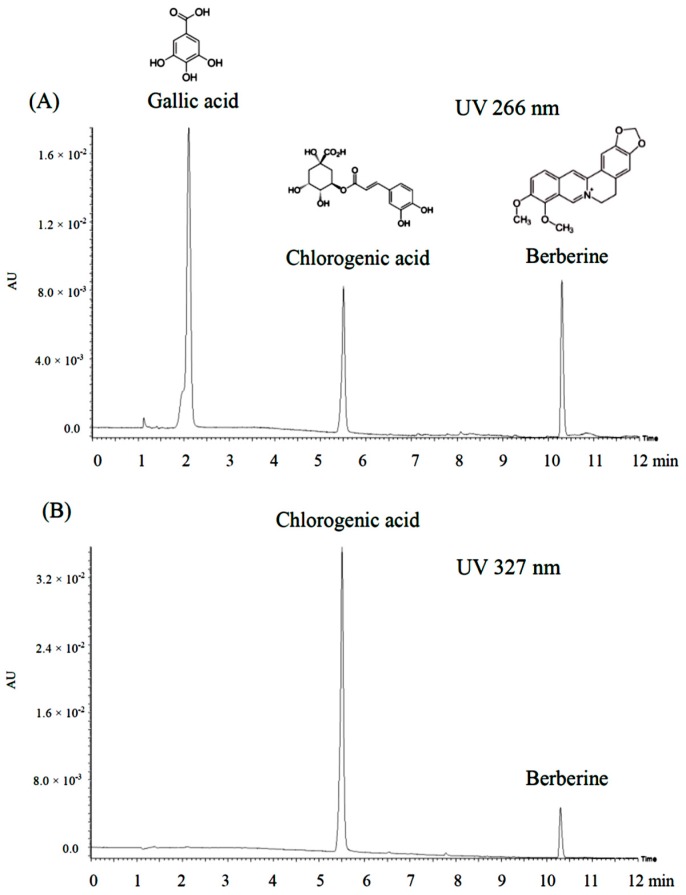

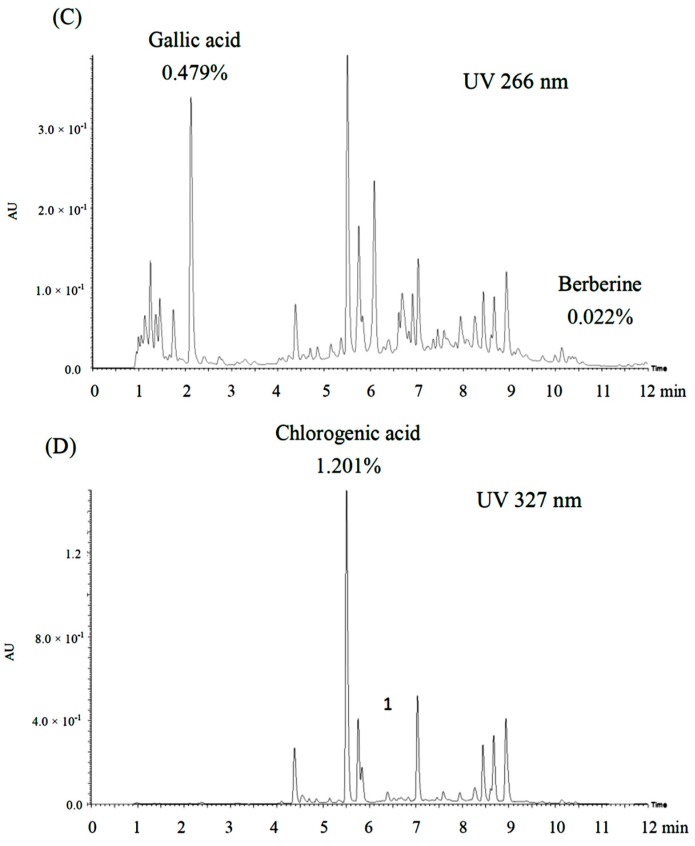
HPLC profiles of: (**A**) a standard mixture consisting of pure compounds Gallic acid, chlorogenic acid and berberine detected at UV 266 nm and (**B**) at UV 327 nm; (**C**) bioactive markers of Gallic acid and berberine in PHF water extract detected at UV 266 nm; and (**D**) bioactive marker of chlorogenic acid in PHF water extract detected at UV 327 nm. Representative retention time peaks and percentage content of the bioactive markers are shown.

The above findings prompted us to investigate the active ingredients involved in the PHF that are responsible for the anti-inflammatory action in the OXA-mediated dermatitis model. Gallic acid (GA), chlorogenic acid (CGA) and berberine (Ber) were identified as the bioactive ingredients or authentication markers for Cortex Moutan, Flos Lonicerae and Cortex Phellodendri, respectively [12,31]. Using chemical analysis, the total content of phenolic compounds in PHF was found to be 4.3%. By comparing the HPLC profiles detected at UV 266 nm, PHF revealed a relatively high peak at retention time 2.0 min, which is the representative time peak of Gallic acid (Figure 7C). Another high peak appeared at retention time 5.5 min at both 266 nm and 327 nm, which is the retention time peak of chlorogenic acid (Figure 7C,D). A small peak was observed at retention time 10.3 min at 266 nm, which is similar to the retention time peak of berberine (Figure 7C). Accordingly, the concentration of Gallic acid, chlorogenic acid and berberine in the PHF water extract was found to be 0.479%, 1.201% and 0.022%, respectively. Since chlorogenic acid is a heat sensitive compound, its concentration in the present study was only 1.201% after boiling, which is much less than the usual yield. Therefore, heating for an extended period of time of PHF may be one of the factors that affects the extraction yield of chlorogenic acid. Yet, the extraction yield may also vary in different herbs. We boiled the PHF for an hour in this study because we intended to mimic the preparation procedures of herbal decoctions of households. The above findings suggested that Gallic acid, chlorogenic acid and berberine may be the active ingredients in the PHF decoction that contribute to the anti-inflammatory action on AD.

### 2.8. In vitro Effects of Berberine on the IL-31- and IL-33-Induced Release of AD-Related Cytokines and Chemokines from Human Eosinophils and Eosinophils-Dermal Fibroblasts Co-Culture

**Figure 8:**
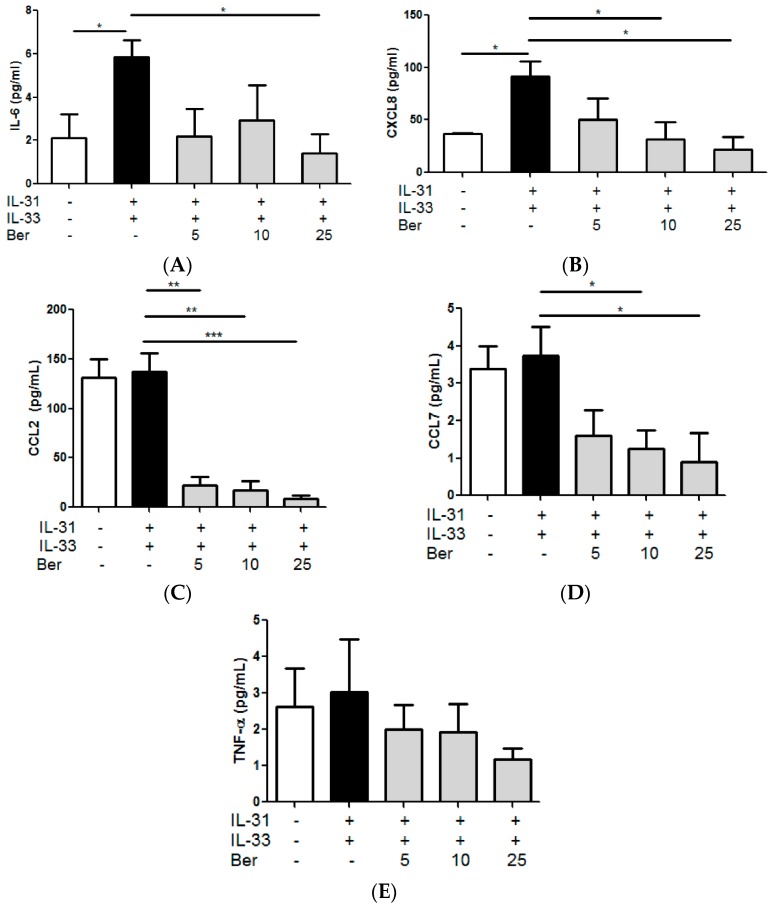
*In vitro* effect of: (**A**) IL-6; (**B**) CXCL8; (**C**) CCL2; (**D**) CCL7; and (**E**) TNF-α release from IL-31- and IL-33-activated human eosinophils treated with berberine (Ber). Eosinophils (3 × 10^5^) was pretreated with or without Ber (5, 10 and 25 µg/mL) and then cultured with or without human recombinant IL-31 and IL-33 (100 ng/mL) for 18 h. Release of cytokines and chemokines in culture supernatants was determined by ELISA and Bio-plex pro assay. Results are shown in bar charts with mean ± SEM (*n* = 4). * *p* < 0.05, ** *p* < 0.01, *** *p* < 0.005 when compared with the IL-31- and IL-33-stimulated group.

**Figure 9:**
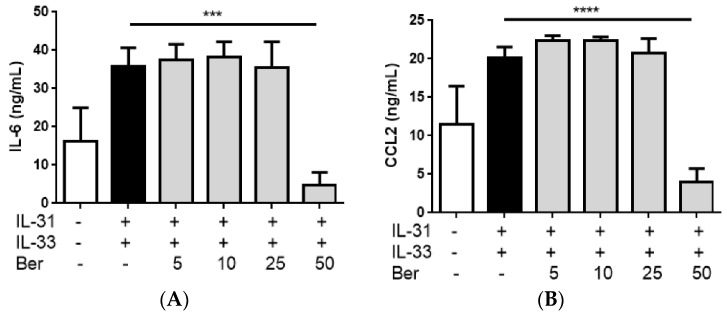

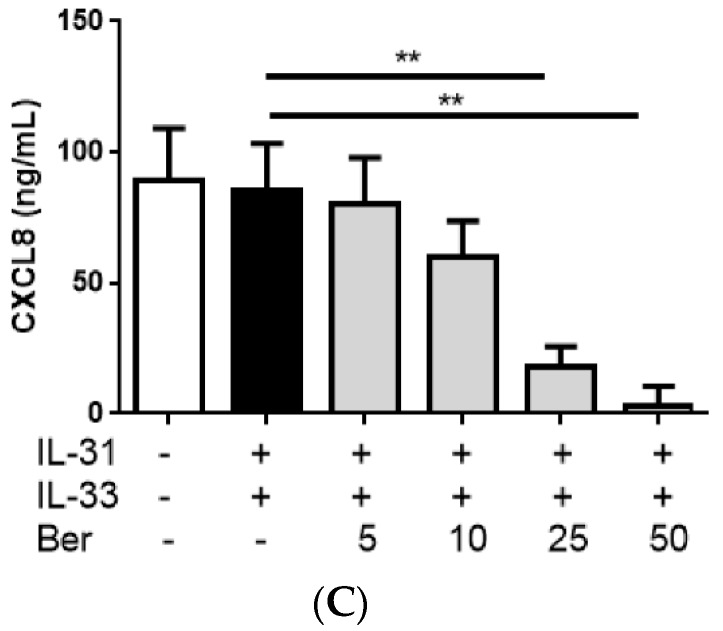
*In vitro* effect of: (**A**) IL-6; (**B**) CCL2; and (**C**) CXCL8 release from IL-31- and IL-33-activated human eosinophils-dermal fibroblasts co-culture treated with berberine (Ber). Eosinophils (3 × 10^5^) and dermal fibroblast (8 × 10^4^) were pre-treated with or without Ber (5, 10, 25 and 50 µg/mL) and then cultured with or without human recombinant IL-31 and IL-33 (100 ng/mL) for further 18 h. Release of AD-related cytokine and chemokines in culture supernatants was determined by ELISA. Results are shown in bar charts with mean ± SEM (*n* = 5). ** *p* < 0.01, *** *p* < 0.0001, **** *p* < 0.00001 compared with IL-31- and-IL-33-stimulated group.

From the reduction of epidermal thickness, eosinophils infiltration and the release of AD-related cytokine in the *in vivo* model, we speculated that the recruitment and activation of eosinophils were significantly suppressed upon the oral or topical treatment of PHF. IL-31 and IL-33 play crucial immunopathological roles in AD [15]. Since human eosinophils constitutively express functional receptor complex for IL-31 and IL-33 receptor ST2, IL-31 and IL-33 were used as an allergic inflammatory inducer in the eosinophils culture system [15]. After incubating berberine (5, 10, 25 µg/mL), one of the potential active ingredients of PHF, with IL-31- and-IL-33-stimulated eosinophils or eosinophils-dermal fibroblasts co-culture for 18 h, the release of pro-inflammatory cytokine IL-6 and AD-related chemokine CXCL8, CCL2 and CCL7, and IL-6, CCL2 and CXCL8, respectively, in the culture supernatant were significantly reduced (Figure 8 and Figure 9, all *p* < 0.05). IL-6 is produced by most innate immune cells including eosinophils as well as non-leukocytes such as epithelial cells and fibroblasts [32,33]. Elevation of IL-6 promotes the production of Th2-related cytokine IL-4 and IL-13, which are the IgE-inducing cytokines that are often associated with AD [32,34]. Upon activation, eosinophils release AD-related chemokine CXCL8 to attract neutrophils; and release CCL2, which would selectively recruit immune cells including monocytes, neutrophils and lymphocytes, and enhances the secretion of IL-4 [18,35]. AD-related CCL7 actively attracts monocytes to the inflammatory sites [36]. Our results therefore show that the activation of eosinophils and subsequent allergic inflammation were suppressed by berberine.

### 2.9. In Vitro Effects of Gallic Acid on the IL-31- and IL-33-Induced Release of AD-Related Cytokines and Chemokines from Human Eosinophils

**Figure 10:**
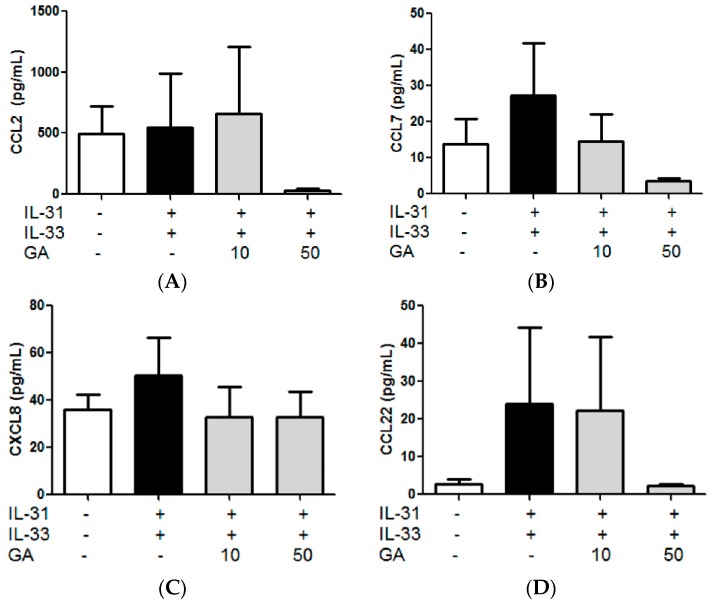

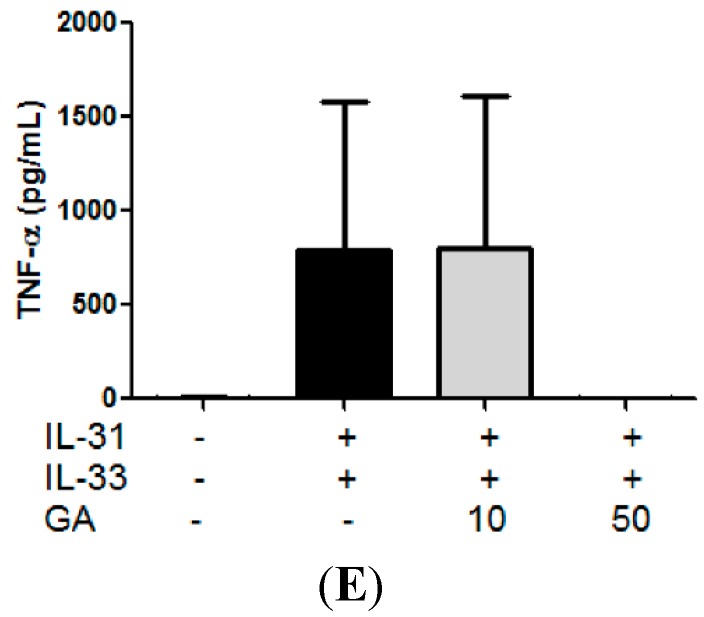
*In vitro* effect of: (**A**) CCL2; (**B**) CCL7; (**C**) CXCL8; (**D**) CCL22; and (**E**) TNF-α release from IL-31- and IL-33-activated human eosinophils treated with Gallic acid (GA). Eosinophils (3 × 10^5^) was pretreated with or without Gallic acid (10 and 50 µg/mL) and then cultured with or without human recombinant IL-31 and IL-33 (100 ng/mL) for further 18 h. Release of cytokines and chemokines in culture supernatants was determined by ELISA and Bio-plex pro assay. Results are shown in bar charts with mean ± SEM (*n* = 4).

Similarly, Gallic acid (10, 50 µg/mL), another potential active ingredients of PHF, were incubated with IL-31- and IL-33-stimulated eosinophils. However, no significant suppression effects were observed on the release of pro-inflammatory cytokines and chemokines tested (Figure 10, all *p* > 0.05).

### 2.10. Effects of Gallic Acid on the IL-31- and IL-33-Induced Release of AD-Related Cytokines and Chemokines from Co-Culture of Eosinophils-Human Dermal Fibroblasts

**Figure 11:**
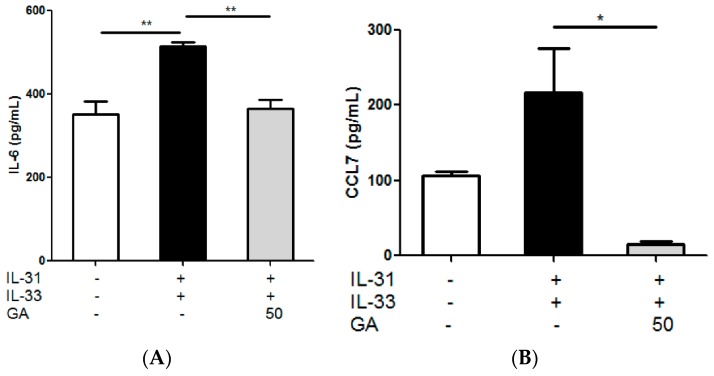

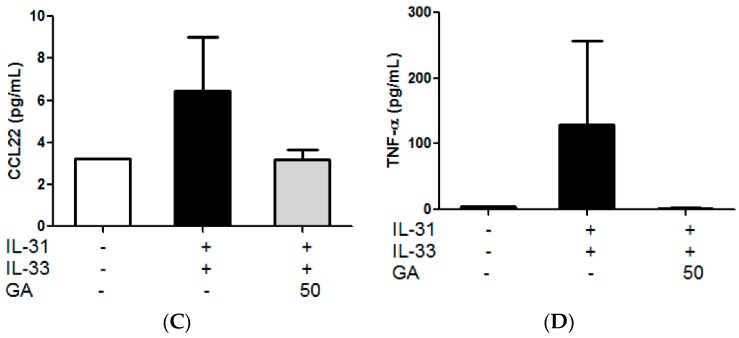
*In vitro* effect of: (**A**) IL-6; (**B**) CCL7; (**C**) CCL22; and (**D**) TNF-α release from IL-31- and IL-33-activated human eosinophils-dermal fibroblast co-culture treated with Gallic acid (GA). Eosinophils (3 × 10^5^) and dermal fibroblasts (8 × 10^4^) were pretreated with or without GA (50 µg/mL) and then cultured with or without human recombinant IL-31 and IL-33 (100 ng/mL) for further 18 h. Release of cytokines and chemokines in culture supernatants was determined by ELISA and Bio-plex pro assay. Results are shown in bar charts with mean ± SEM (*n* = 3). * *p* < 0.05, ** *p* < 0.01 when compared with the IL-31- and IL-33-stimulated group.

By incubating Gallic acid (50 µg/mL) in the co-culture of eosinophils with dermal fibroblasts, the release of pro-inflammatory cytokine IL-6 and the chemokine CCL7 in the culture supernatant were significantly reduced (Figure 11, *p* < 0.05). This further supported that active ingredient Gallic acid could exhibit anti-inflammatory activities upon the interaction between eosinophils and dermal fibroblasts in AD. Further mechanistic studies are required to elucidate the signaling mechanism involved.

**Figure 12:**
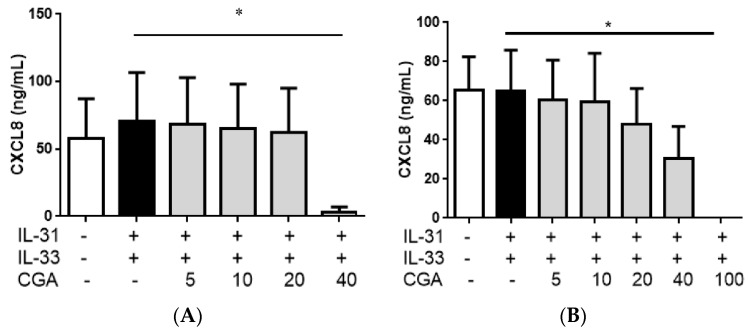
*In vitro* effect of CXCL8 release from IL-31- and IL-33-activated (**A**) eosinophils single alone and (**B**) eosinophils-dermal fibroblasts co-culture treated with chlorogenic acid (CGA). Eosinophils (3 × 10^5^) with or without co-culture with dermal fibroblast (8 × 10^4^) were pre-treated with or without CGA (5, 10, 20 and 40 µg/mL) and then cultured with or without human recombinant IL-31 and IL-33 (100 ng/mL) for further 18 h. Release of CXCL8 in culture supernatants was determined by ELISA. Results are shown in bar charts with mean ± SEM (*n* = 4). * *p* < 0.05 when compared IL-31- and-IL-33-stimulated group.

Chlorogenic acid is another ingredient found in the PHF water extract. Chlorogenic acid is a common form of phenolic compound found in plants, and has been widely reported to possess both *in vitro* and *in vivo* anti-inflammatory potential in allergic diseases [37,38,39]. It has been shown that chlorogenic acid, active ingredient of Flos Lonicerae, could attenuate eosinophilia, reduce the serum IgE level and Th2 cytokine production in asthmatic mouse model [37]. Its underlying anti-allergic mechanism has also been elucidated previously [37]. As shown in Figure 12, chlorogenic acid could significantly suppress the release of AD-related chemokine CXCL8 release from eosinophils single culture or eosinophils-dermal fibroblasts co-culture.

Previous studies has shown that herbal medicine can generally be absorbed to the skin through transdermal drug diffusion, and Pentaherbs formula can be detected in the porcine ear skin in the previous transdermal drug permeation tests [40,41]. In the present study, PHF was directly applied on the ears of the mice, which have less hair, and contain abundant of blood vessels that facilitate the absorption of herbs. Moreover, in the single- and multiple-OXA-challenged mice models, the major physical barrier of the skin, *i.e.*, stratum corneum, was damaged due to the scratching behavior of the mice. With the help of cream vehicle, these three PHF active ingredients may be able to penetrate the damaged skin to exert its anti-atopic and anti-inflammatory activity in the skin or in the immune system. Together, all HLPC identified bioactive compounds Gallic acid, berberine and chlorogenic acid should contribute to the *in vivo* anti-inflammatory activities of PHF in AD.

Following the previous clinical study of PHF showing the clinically beneficial and effective outcome in AD patients [7], the main objective of this study is to try to further evaluate the *in vivo* anti-inflammatory activity of PHF complex formula in dermatitis mice. Regarding the *in vivo* study of the inhibitory effects of Gallic acid, chlorogenic acid and berberine, it is speculated that the anti-inflammatory activities of these small size compounds may be lower than that of PHF. It is because these small compounds may require other ingredients and components of PHF to further enhance their intestinal absorption and bioavailability in mice. Because of the above, in the present study, we prefer to use PHF to evaluate its anti-inflammatory activities in mice with OXA-mediated dermatitis in order to mimic its clinical applications in human AD.

## 3. Experimental Section

### 3.1. Endotoxin-Free Solutions

Cell culture medium was purchased from Gibco Invitrogen Corp, Carlsbad, CA, USA, free of detectable lipopolysaccharide (LPS, <0.1 EU/mL). All other solutions were prepared using pyrogen-free water and sterile polypropylene plasticware. No solution contained detectable LPS, as determined by the Limulus amoebocyte lysate assay (sensitivity limit 12 pg/mL; Biowhittaker Inc., Walkersville, MD, USA).

### 3.2. Water Extraction of Pentaherbs Formula (PHF)

The five herbal components of PHF, including 5 g Flos Lonicerae (Jinyinhua), 2.5 g Herba Menthae (Bohe), 5 g Cortex Moutan (Danpi/DP), 5 g Rhizoma Atractylodis (Cangzhu), and 5 g Cortex Phellodendri (Huangbai) were purchased and extracted by refluxing in boiling water at 100 °C for 1 h. Extraction was repeated twice to obtain total water crude extract. The filtered extract were freeze-dried into powder and stored in desiccator. These processes fulfilled the Good Manufacturing Practice according to the Australian Therapeutic Goods Administration standard [13].

### 3.3. Mice

Inbred adult female BALB/c mice (8 week old, 20 g body weight) were purchased from The Laboratory Animal Services Centre, The Chinese University of Hong Kong (Hong Kong, China). All animal experiments were conducted in accordance with the principles outlined in the Animal Experimentation Ethics Committee Guide for the Care and Use of Laboratory Animals, as approved by the Animal Experimentation Ethics Committee of the Chinese University of Hong Kong.

### 3.4. Single Challenge Oxazolone (OXA)-Induced Dermatitis-Like Mice Model

Oxazolone-induced dermatitis-like mice model were established according to Traidl *et al.* with slight modifications [42]. Briefly, the dorsal sides of the mice were topically sensitized with 1% oxazolone (Sigma Chemical Co., St. Louis, MO, USA), or vehicle (acetone, Sigma) after being shaved using hair-removal cream (Reckitt Benckiser Group, Parsippany, NJ, USA) on Day 1. The right ear of the mice was then topically challenged with 1% oxazolone, while the left ears was treated with acetone on Day 7. On Day 9, the ear thicknesses were measured using a dial thickness gauge (Model G, Peacock, OZAKI MFG. Co., Ltd., Tokyo, Japan). Ear swelling of each mouse was calculated by subtracting the ear thickness of the left ear from that of the right ear. Photographs of the mice ears were taken before the mice were euthanized. Serum of each mouse was collected for cytokines and chemokines determination.

### 3.5. Multiple Oxazolone (OXA)-Induced AD-Like Mice Model

Oxazolone-induced AD-like mice model were established according to Man *et al.* with slight modifications [27]. The dorsal sides of the shaved mice were topically sensitized with 200 µL of 1% oxazolone, or acetone on Day 1. After a week, the right ear of the mice was then topically challenged with 20 µL of 1% oxazolone; while the left ear was treated with 20 µL acetone every other day for ten times. Vehicle cream alone, which is composed of wool fat and Vaseline in 1:20 ratio, or Pentaherbs formula (50 mg/mL) in cream vehicle, were topically applied to both ears of the mice from Day 1 to Day 25 every other day. Dexamethasone (10 µg/ear) was applied topically on mouse ears as positive control from Day 16 to Day 26 thirty minutes before challenge. Ear thicknesses were measured using a dial thickness gauge three hours before each challenge or treatment. Ear swelling of each mouse was calculated by subtracting the ear thickness of the left ear from that of the right ear. Photographs of the mice ears were taken before the mice were sacrificed on Day 27. Serum of each mouse was collected for cytokines determination.

### 3.6. Histological Examination

Ear tissues were excised and fixed in 4% paraformaldehyde overnight. They were then dehydrated and embedded in paraffin. Paraffin sections (4 µm) were cut and stained by Hematoxylin & Eosin (H & E) (Beyotime Institute of Biotechnology, Jiangsu, China) for standard histopathological observation [43]. Mast cells were stained using Toluidine blue (Sigma) for the determination of mast cells infiltration. The thickness of the epidermis was measured from the stratum corneum to the basal membrane using software ImageJ (U.S. National Institutes of Health, USA). The number of eosinophils infiltrated into the epidermal and dermal layers was determined by counting three random fields per specimen at 400× magnification using Leica DM6000B microscope (Leica Microsystems GmbH, Wetzlar, Germany) and processed using the Leica Application Suite software (Leica Microsystems GmbH); while the number of mast cells infiltrated into the dermal layer was determined by counting three random fields per specimen at 200× magnification. The number of eosinophils infiltrated per mm^2^ were then calculated.

### 3.7. Chemical Analysis of Total Phenolic Compounds

The total phenolic compounds in PHF was analyzed using chemical methods with Folin-Ciocalteu’s phenol reagent [44].

### 3.8. Identification of Active Ingredients from Pentaherbs Formula Using High Pressure Liquid Chromatography (HPLC)

The presence of Gallic acid, berberine, and chlorogenic acid in the Pentaherbs formula were identified and quantified using HPLC analysis [12,13]. HPLC were performed using Waters ACQUITY Uultra Performance Liquid Chromatography (UPLC) System (Waters Corp., Milford, MA, USA). PHF was dissolved in double distilled water and sample solution was injected onto an Agilent Poroshell 120 EC-C18 column (100 × 4.6 mm) (Agilent Technologies, Santa Clara, CA, USA), equipped with Agilent Ultra High Performance Liquid Chromatography (UHPLC) Guard Poroshell 120 EC-C18 guard column (5 × 4.6 mm) (Agilent Technologies). All solvents were pre-filtered with 0.45 µm Millipore filter discs (Millipore, Temecula, CA, USA) and de-gassed. A gradient elution was carried out using the following solvent system: mobile phase A—acetonitrile; mobile phase B—0.1% acetic acid in water. The flow rate was 1.0 mL/min and detection was performed at 266 nm and 327 nm. Each sample (5 µL) was injected into the column. Identification of bioactive markers was carried out by comparing the retention times and the UV absorbance of the unknown peaks to those of the standards. Standard Gallic acid (1 mg/mL), berberine (1 mg/mL) and chlorogenic aicd (1 mg/mL) were prepared and analyzed.

### 3.9. Purification of Human Blood Eosinophils

Fresh buffy coat obtained from healthy volunteer human blood donors of Hong Kong Red Cross Blood Transfusion Service were diluted with cold PBS in 1:2, and centrifuged using an isotonic Percoll solution (density 1.082 g/mL; Amersham and Pharmacia Biotech, Uppsala, Sweden) for 23 min at 2300 rpm. The eosinophil-rich granulocytes fraction was collected and washed with cold 2% fetal bovine serum (FBS) in PBS twice. The cells were then incubated with anti-CD16 magnetic microbeads (Miltenyi Biotec, Bergisch Gladbach, Germany) at 4 °C for 30 min with constant rotation. CD16-positive eosinophils were then collected from the flow-through fraction of the LS+ column (Miltenyi Biotec, Bergisch Gladbach, Germany) during the depletion under the magnetic field. The purity of isolated eosinophils was assessed by Hemacolor rapid blood smear stain (E Merck Diagnostica, Darmstadt, Germany). Eosinophils with >90% purity were cultivated in RPMI 1640 medium (Gibco) supplemented with 10% heat-inactivated-FBS. The above protocol using human eosinophils purified from human buffy coat was approved by the Clinical Research Ethics Committee of The Chinese University of Hong Kong-New Territories East Cluster Hospitals.

### 3.10. Eosinophils Culture and Co-Culture of Eosinophils and Dermal Fibroblasts

The human primary dermal fibroblasts (HDF) were purchased from Life Technologies, Carlsbad, CA, USA and were cultivated in Medium 106 (Gibco) supplemented with low serum growth supplement (Thermo Fisher Scientific) and antibiotic antimycotic solution (Gibco) in cell culture flasks at 37 °C in a 5% CO_2_, 95% humidified air until confluence to cell monolayer. In the co-culture system, RPMI1640 medium supplemented with 10% FBS were used in place of Medium 106. Eosinophils (3 × 10^5^ cells) with or without dermal fibroblasts (8 × 10^4^ cells) were pre-treated with or without Gallic acid or berberine, and were then incubated with 100 ng/mL IL-31 (R & D Systems, Minneapolis, MN, USA) and 100 ng/mL IL-33 (R & D Systems, Minneapolis, MN, USA) for further 18 h.

### 3.11. Measurement of Cytokines/Chemokines

Concentrations of human pro-inflammatory cytokine IL-6 and chemokine CXCL8 release in culture supernatant and mouse IL-4 were determined using ELISA kits purchased from Biolegend Inc., San Diego, CA, USA. Concentrations of CCL2, CCL7, CCL22 and tumor necrosis factor (TNF)-α released in culture supernatant, and the mouse IL-5, keratinocytes chemoattractants (KC), IL-6, macrophage inflammatory protein 2 (MIP-2) released in the mouse serum were quantitated with Multiplex MAP kit (EMD Millipore Corp., Billerica, MA, USA) and Bio-plex pro assay kit reagents using Luminex Bio-plex 200 suspension array system (Bio-Rad Corp. Hercules, CA, USA).

### 3.12. Measurement of Murine Serum Total IgE

Murine serum total IgE concentration was measured using ELISA kit (Biolegend).

### 3.13. Data Analysis

All experiments were performed at least three times. Results were analyzed using Student’s *t*-test for comparisons using GraphPad PRISM software version 5.0 (GraphPad Software, San Diego, CA, USA). A significant level with *p* < 0.05 was considered significantly different.

## 4. Conclusions

Oral administration or topical treatment of Pentaherbs formula could ameliorate inflammation of oxazolone-mediated dermatitis mice by reducing epidermis thickening, eosinophils and mast cells infiltration and the release of pro-inflammatory cytokine IL-12 (p70) and eosinophil activator IL-5. Gallic acid, berberine and chlorogenic acid are the active ingredients in the PHF water extracts, which are involved in the suppression of the activation of eosinophils, the principal effector cells in allergic inflammation. The current study provided further scientific evidence to support our previous clinical study that Pentaherbs formula could potentially be an alternative adjunct therapy for the treatment of AD. Further investigation of the Gallic acid, chlorogenic acid and berberine-mediated intracellular signal transduction mechanism in eosinophils upon interacting dermal fibroblasts in AD is ongoing.

## References

[1] Totri C.R., Diaz L., Eichenfield L.F. (2014). 2014 update on atopic dermatitis in children. Curr. Opin. Pediatr..

[2] Wong G.W., Hui D.S., Chan H.H., Fok T.F., Leung R., Zhong N.S., Chen Y.Z., Lai C.K. (2001). Prevalence of respiratory and atopic disorders in Chinese schoolchildren. Clin. Exp. Allergy.

[3] Wong G.W., Leung T.F., Ko F.W. (2013). Changing prevalence of allergic diseases in the Asia-pacific region. Allergy Asthma Immunol. Res..

[4] Williams H.C. (2005). Clinical practice. Atopic dermatitis. N. Engl. J. Med..

[5] Asher M.I., Montefort S., Björkstén B., Lai C.K., Strachan D.P., Weiland S.K., Williams H., ISAAC Phase Three Study Group (2006). Worldwide time trends in the prevalence of symptoms of asthma, allergic rhinoconjunctivitis, and eczema in childhood: ISAAC phases one and three repeat multicountry cross-sectional surveys. Lancet.

[6] Boguniewicz M., Leung D.Y. (2011). Atopic dermatitis: A disease of altered skin barrier and immune dysregulation. Immunol. Rev..

[7] Hon K.L., Leung T.F., Ng P.C., Lam M.C., Kam W.Y., Wong K.Y., Lee K.C., Sung Y.T., Cheng K.F., Fok T.F. (2007). Efficacy and tolerability of a Chinese herbal medicine concoction for treatment of atopic dermatitis: A randomized, Double-blind, Placebo-controlled study. Br. J. Dermatol..

[8] Chan B.C., Hon K.L., Leung P.C., Sam S.W., Fung K.P., Lee M.Y., Lau H.Y. (2008). Traditional Chinese medicine for atopic eczema: PentaHerbs formula suppresses inflammatory mediators release from mast cells. J. Ethnopharmacol..

[9] Ow Y.Y., Stupans I. (2003). Gallic acid and gallic acid derivatives: Effects on drug metabolizing enzymes. Curr. Drug Metab..

[10] Faried A., Kurnia D., Faried L.S., Usman N., Miyazaki T., Kato H., Kuwano H. (2007). Anticancer effects of gallic acid isolated from Indonesian herbal medicine, *Phaleria macrocarpa* (Scheff.) Boerl, on human cancer cell lines. Int. J. Oncol..

[11] Kaur M., Velmurugan B., Rajamanickam S., Agarwal R., Agarwal C. (2009). Gallic acid, an active constituent of grape seed extract, exhibits anti-proliferative, pro-apoptotic and anti-tumorigenic effects against prostate carcinoma xenograft growth in nude mice. Pharm. Res..

[12] Chan B.C., Li L.F., Hu S.Q., Wat E., Wong C.W., Zhang V.X., Lau C.B., Wong C.K., Hon K.L., Hui P.C. (2015). Gallic Acid is the major active component of Cortex Moutan in inhibiting immune maturation of human monocyte-derived dendritic cells. Molecules.

[13] Liu K.Y., Hu S., Chan B.C., Wat E.C., Lau C.B., Hon K.L., Fung K.P., Leung P.C., Hui P.C., Lam C.W. (2013). Anti-inflammatory and anti-allergic activities of Pentaherbs formula, Moutan Cortex (Danpi) and gallic acid. Molecules.

[14] Kim B.Y., Park H.R., Jeong H.G., Kim S.W. (2015). Berberine reduce allergic inflammation in a house dust mite allergic rhinitis mouse model. Rhinology.

[15] Wong C.K., Leung K.M., Qiu H.N., Chow J.Y., Choi A.O., Lam C.W. (2012). Activation of eosinophils interacting with dermal fibroblasts by pruritogenic cytokine IL-31 and alarmin IL-33: Implications in atopic dermatitis. PLoS ONE.

[16] Scanlon S.T., McKenzie A.N. (2012). Type 2 innate lymphoid cells: New players in asthma and allergy. Curr. Opin. Immunol..

[17] Valent P. (2009). Interleukin-33: A regulator of basophils. Blood.

[18] Cheung P.F., Wong C.K., Ho A.W., Hu S.Q., Chen D.P., Lam C.W. (2010). Activation of human eosinophils and epidermal keratinocytes by Th2 cytokine IL-31: Implication for the immunopathogenesis of atopic dermatitis. Int. Immunol..

[19] Simon D., Braathen L.R., Simon H.U. (2004). Eosinophils and atopic dermatitis. Allergy.

[20] Bruijnzeel P.L., Kuijper P.H., Kapp A., Warringa R.A., Betz S., Bruijnzeel-Koomen C.A. (1993). The involvement of eosinophils in the patch test reaction to aeroallergens in atopic dermatitis: Its relevance for the pathogenesis of atopic dermatitis. Clin. Exp. Allergy.

[21] Matsuda H., Watanabe N., Geba G.P., Sperl J., Tsudzuki M., Hiroi J., Matsumoto M., Ushio H., Saito S., Askenase P.W. (1997). Development of atopic dermatitis-like skin lesion with IgE hyperproduction in NC/Nga mice. Int. Immunol..

[22] Kiehl P., Falkenberg K., Vogelbruch M., Kapp A. (2001). Tissue eosinophilia in acute and chronic atopic dermatitis: A morphometric approach using quantitative image analysis of immunostaining. Br. J. Dermatol..

[23] Uehara M., Izukura R., Sawai T. (1990). Blood eosinophilia in atopic dermatitis. Clin. Exp. Dermatol..

[24] Taniuchi S., Chihara J., Kojima T., Yamamoto A., Sasai M. (2001). Serum eosinophil derived neurotoxin may reflect more strongly disease severity in childhood atopic dermatitis than eosinophil cationic protein. J. Dermatol. Sci..

[25] Jenerowicz D., Czarnecka-Operacz M., Silny W. (2006). Selected eosinophil proteins as markers of inflammation in atopic dermatitis patients. Acta Dermatovenerol. Croat..

[26] Schröder J.M., Mochizuki M. (1999). The role of chemokines in cutaneous allergic inflammation. Biol. Chem..

[27] Man M., Hatano Y., Lee S.H., Man M., Chang S., Feingold K.R., Leung D.Y.M., Holleran W., Uchida Y., Elias P.M. (2008). Characterization of a hapten-induced, murine model with multiple features of atopic dermatitis: Structural, immunologic, and biochemical changes following single versus multiple oxazolone challenges. J. Investig. Dermatol..

[28] Oyoshi M.K., He R., Kanaoka Y., ElKhal A., Kawamoto S., Lewis C.N., Austen K.F., Geha R.S. (2012). Eosinophil-derived leukotriene C4 signals via type 2 cysteinyl leukotriene receptor to promote skin fibrosis in a mouse model of atopic dermatitis. Proc. Natl. Acad. Sci. USA.

[29] Grewe M., Czech W., Morita A., Werfel T., Klammer M., Kapp A., Ruzicka T., Schӧpf E., Krutmann J. (1998). Human eosinophils produce biologically active IL-12: Implications for control of T cell responses. J. Immunol..

[30] Yawalkar N., Karlen S., Egli F., Brand C.U., Graber H.U., Pichler W.J., Braathen L.R. (2000). Down-regulation of IL-12 by topical corticosteroids in chronic atopic dermatitis. J. Allergy Clin. Immunol..

[31] Chinese Pharmacopoeia Commission, China (2010). Chinese Pharmacopoeia.

[32] Rincon M. (2012). Interleukin-6: From an inflammatory marker to a target for inflammatory diseases. Trends Immunol..

[33] Moqbel R., Levi-Schaffer F., Kay A.B. (1994). Cytokine generation by eosinophils. J. Allergy Clin. Immunol..

[34] Tazawa T., Sugiura H., Sugiura Y., Uehara M. (2004). Relative importance of IL-4 and IL-13 in lesion skin of atopic dermatitis. Arch. Dermatol. Res..

[35] Deshmane S.L., Kremlev S., Amini S., Sawaya B.E. (2009). Moocyte chemoattractant protein-1 (MCP-1): An overview. J. Interferon Cytokine Res..

[36] Romagnani S. (2002). Cytokines and chemoattractants in allergic inflammation. Mol. Immunol..

[37] Kim H.R., Lee D.M., Lee S.H., Seong A.R., Gin D.W., Hwang J.A., Park J.H. (2010). Chlorogenic acid suppresses pulmonary eosinophilia, IgE production, and Th2-type cytokine production in an ovalbumin-induced allergic asthma: Activation of STAT-6 and JNK is inhibited by chlorogenic acid. Int. Immunopharmacol..

[38] Krakauer T. (2002). The polyphenol chlorogenic acid inhibits staphylococcal exotoxin-induced inflammatory cytokines and chemokines. Immunopharmacol. Immunotoxicol..

[39] Hwang S.J., Kim Y.W., Park Y., Lee H.J., Kim K.W. (2014). Anti-inflammatory effects of chlorogenic acid in lipopolysaccharide-stimulated RAW 264.7 cells. Inflamm. Res..

[40] Yun Y., Lee S., Kim S., Choi I. (2013). Inpatient treatment for severe atopic dermatitis in a traditional Korean Medicine hospital: Introduction and retrospective chart review. Complement. Ther. Med..

[41] Wang W., Wat E., Hui C.L., Ng S.F., Kan C.W., Wong C.W., Chan B., Lau B.S., Leung P.C. (2015). Application of Chinese herbal medicine onto cotton fabric by dyeing methods. Fibers Polym..

[42] Traidl C., Jugert F., Krieg T., Merk H., Hunzelmann N. (1999). Inhibition of allergic contact dermatitis to DNCB but not to oxazolone in interleukin-4-deficient mice. J. Investig. Dermatol..

[43] Jiao D., Wong C.K., Qiu H.N., Dong J., Cai Z., Chu M., Hon K.L., Tsang M.S., Lam C.W. (2015). NOD2 and TLR2 ligands trigger the activation of basophils and eosinophils by interacting with dermal fibroblasts in atopic dermatitis-like skin inflammation. Cell Mol. Immunol..

[44] Anesini C., Ferraro G.E., Filip R. (2008). Total polyphenol content and antioxidant capacity of commercially available tea (*Camellia sinensis*) in Argentina. J. Agric. Food Chem..

